# Prevalence of Psychological Stress Among Women Attending the Assisted Reproductive Technology (ART) Unit at King Abdulaziz Medical City: A COVID-19 Pandemic Experience

**DOI:** 10.7759/cureus.47145

**Published:** 2023-10-16

**Authors:** Samaher Alfaraj, Asem Alfagih, Eman F Al-Zahrani, Ghadeer L Aljahdali, Hayat Alrabieaa, Fahad Alsalman, Rashpal Gill, Bayan Albadah

**Affiliations:** 1 In-Vitro Fertilization (IVF) Unit, King Abdulaziz Medical City, Riyadh, SAU; 2 Urogynecology and Reconstructive Pelvic Surgery, King Abdulaziz Medical City, Riyadh, SAU; 3 Medicine, King Saud Bin Abdulaziz University for Health Sciences, Riyadh, SAU; 4 Biostatisitcs, King Abdullah International Medical Research Center, Riyadh, SAU

**Keywords:** infertility, assisted reproductive technology, in-vitro fertilization, covid-19, psychological stress

## Abstract

Aim

The present study aimed to measure the prevalence of psychological stress among women attending the Assisted Reproductive Technology (ART) unit in King Abdulaziz Medical City during the COVID-19 pandemic.

Method

The study adopted a descriptive cross-sectional design and was conducted between January 2020 and December 2022. A sample of 104 women attending the ART unit were recruited in this study. To collect data, the researchers developed a study questionnaire that consisted of 26 items. Nine items were designed to measure the participants’ demographic characteristics, and 17 items were designed to depict their responses related to psychological stress during the COVID-19 pandemic. Data were analyzed using the Statistical Product and Service Solutions (SPSS) (v. 26l; IBM SPSS Statistics for Windows, Armonk, NY). Chi-square and t-tests were used to assess the association between stress and sociodemographics.

Results

The findings of the study showed an overall prevalence of 86.53% (n=90). Levels of psychological stress were moderate (34.62%, n=36), severe (39.42%, n=41), and extremely severe (12.50%, n=13). The study found that there is a significant statistical interaction between the number of previous ART attempts and psychological stress (p=0.0019).

Conclusion

The study concluded that women attending the ART unit at King Abdulaziz Medical City (KAMC), Riyadh, Saudi Arabia, were experiencing high levels of psychological stress, and these levels were affected by the number of previous ART attempts.

## Introduction

The European Society for Human Reproduction and Embryology (ESRHE) and the American Society for Reproductive Medicine (ASRM) recommended withholding all assisted reproductive techniques services during COVID-19 [1]. This cessation of fertility treatment leads to additional stress on couples suffering from infertility for a long period of time and looking forward to conceiving [2]. Stress is a major problem in many countries whether it is physical, social, or psychological. Multiple studies have investigated the psychological effect of this virus on infertile women who are going or planning to go through with ART [3]. One study found that psychological stress, such as anxiety disorder or depression, affects 30% of women who attend infertility clinics, possibly due to infertility diagnosis alone; nonetheless, societal pressures, testing, diagnosis, treatments, failures, unfulfilled desires, and even financial costs, which it is associated with it, should not to be neglected [4].

Furthermore, this global pandemic had new stressors associated with it, such as strict social distancing, negative effects of media, self-isolation, lack of physical activity and emotional connections, and home isolation [5]. As a result of these new stressors, a greater impact on the individual’s mental health and well-being has been shown, especially in infertile couples where this pandemic added a burden on top of the infertility stress itself [6].

Recently, numerous types of research and studies have highlighted how psychological stressors and their effect on psychological well-being may lead to fewer chances of conceiving via ART. However, the specific pathways or mechanisms regarding how stress affects these outcomes have not been conclusively identified. Most studies suggested that anxiety and depression negatively affect sex hormones and neuroendocrine or immunologic functions related to pregnancy [7].

A cross-sectional study found that 72% of participating couples diagnosed with a psychological illness (e.g., stress, anxiety, and depression in the respective partner) were found to have a lower likelihood of clinical pregnancy and live birth. One theory suggests that this could be attributed to a lower serum TGF‐β and higher cervicovaginal IL‐6 and IL‐1β that are associated with stress. In that same study, it was suggested that depression and stress caused an increase in cytokines in both partners, which was indicative of in-vitro fertilization (IVF) failure [8].

Another study suggested that, as a result of stress, there is a decrease in GnRH and gonadotropin secretion, and this is attributed to an alteration in the activity of some stress-related factors. For example, hypothalamic corticotropin-releasing hormone (CRH), which is the principal driving factor of the HPA axis during stress, is a potent inhibitor of GnRH secretion [9]. Some studies suggested that the stress-induced suppression of the HPG axis is attributed to pro-inflammatory cytokines (i.e., interleukin-1β and tumor necrosis factor-α) [9].

Furthermore, the increase in both release/concentration of glucocorticoids was linked to a marked disorder of the hypothalamic-pituitary-ovary (HPO) axis [10]. One study conducted on sheep found that infusing high concentrations of cortisol comparable to those in humans under stress generates a delay in follicular maturation and ovulation by attenuating or blocking the expected increase of estrogen and luteinizing hormone (LH) surge [11].

As highlighted above, stress can decrease the success rate of IVF cycles and conception rate due to multiple factors. For that stress-coping mechanisms, support systems and behavioral therapy should be implemented as it might make a significant difference for women undergoing assisted reproductive techniques for infertility. Women who undergo counseling and seek support may reduce their anxiety and depression levels; therefore, they might increase their chances of becoming pregnant. Positive moods are correlated with increased chances of delivering a live baby, while higher levels of anxiety may increase the chances of stillbirth [12].

One study found that there was a higher conception rate (55%) in women who were a part of cognitive behavioral intervention groups and in those who were part of support groups (54%) in comparison to only (20%) conception rate in women not receiving any intervention [12]. The studies reviewed above were recent studies during the COVID-19 pandemic, and still more studies are ongoing. Hereby, we shed light on psychological stress prevalence among women enrolled in the Assisted Reproductive Technology (ART) unit at King Abdulaziz Medical City (KAMC), Riyadh, Saudi Arabia, during the COVID-19 pandemic due to the importance of stress levels and its effect on success rates.

## Materials and methods

Study design, area, and setting

This is a cross-sectional study in which the prevalence of psychological stress among women seeking IVF treatment is measured. The present study took place in an In-Vitro Fertilization (IVF) Clinic at KAMC, Riyadh, Saudi Arabia, from January 2020 to December 2022. The IVF unit at KAMC was established in October 1995, and the growth in the number of patients and services increased dramatically. The department serves the National Guard employees and their families, as well as referred patients from across the country who meet the admission criteria. The service includes assessment and evaluation of infertility cases and treatment of female and male infertility through assisted reproductive techniques: intra-uterine insemination (IUI), in-vitro fertilization, intra-cytoplasmic sperm injection (ICSI), etc. This study was approved by the Institutional Board Review (IRB) at King Abdullah International Medical Center (KAIMRC) with protocol number: NRC21R/128/03.

Study participants, sample size, and technique

**Figure 1:**
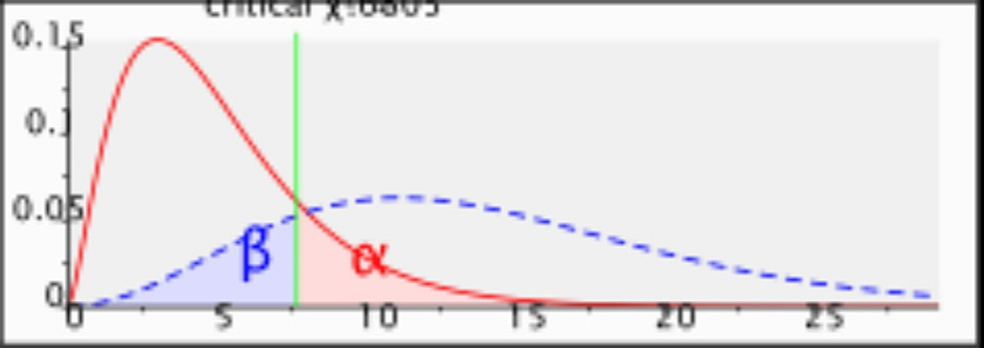
Sample size calculation

The study participants were all women enrolled in the IVF unit at KAMC, Riyadh, Saudi Arabia. Based on the official statistics released by the IVF Unit at KAMC-RD, the average number of IVF cases handled each year was 600. The targeted population was women who either started a new IVF cycle or had started a cycle but were put on hold due to precautionary measures taken during the COVID-19 pandemic. The convenient sampling technique (non-purposive) was used in this study to recruit the study participants. The unit has its own inclusion criteria, including 40 years old or less; no children or only one child; all comorbidities such as hypothyroidism, diabetes mellitus, and hypertension to be controlled; and BMI less than 30 kg/m^2^. To calculate the sample size, we used G*Power (v. 3.9.2.7; Institute for Experimental Psychology, Dusseldorf, Germany) with a medium size effect of 0.3, a non-centrality parameter of 9.00, a degree of freedom of 5, a critical χ2 of 7.6805, it was found that a sample of 100 participants is needed for this study (Figure 1).

Data collection

The study used a researcher-developed questionnaire that consisted of two parts. The first part included nine items to measure data about the participants' age, educational level, cause of infertility, pregnancy, ART attempts, last IVF cycle, and COVID-19 infection. However, the second part involved 17 items that aimed to identify the level of psychological stress among infertile women. These items were scored using the binary system (Yes=1, No=0). A score ranging between 0 and 4.25 was considered mild stress, a score of 4.26-8.51 represented moderate stress, a score of 8.52-12.77 represented severe stress, and a score higher than 12.77 represented extremely severe stress. The questionnaire was validated by four experts (two research experts and two specialty experts) who checked the content of the questionnaire, its appropriateness, and linguistic integrity. To check the reliability of the study questionnaire, a pilot sample of 25 women from the study population was recruited, and the reliability was calculated through Cronbach’s alpha coefficient. It was found that the reliability coefficient value was 0.79, which is an acceptable reliability value in healthcare research. The questionnaire was self-administered to all women attending their appointments in the unit and given appropriate time to finish it individually.

Data analysis

Data were analyzed using the Statistical Package of Social Sciences (SPSS) (v. 26; IBM Corp, Chicago, IL). Descriptive statistics were used to quantitatively describe the study participants’ socio-demographic characteristics and their responses to the study scale. The chi-square test was used to identify the interaction between the participants’ socio-demographic characteristics and the level of psychological stress retrieved from the study scale. A value of (α≤0.05) was used as a significant statistical threshold.

## Results

A total of 104 women attending the IVF clinic were recruited in this study. The results shown in Table 1 represent the socio-demographic and clinical characteristics of all the women. The results showed that women aged 18-24, 25-31, 32-39, and >40 years accounted for 6.73% (n=7), 34.61% (n=36), 47.12% (n=49), and 11.54% (n=12), respectively. The majority of the sample were college graduates, 55.77% (n=58). Unexplained infertility was the most common cause, accounting for 43.77% (n=45). Half of the sample had been pregnant previously, and only 31.73% (n=33) conceived naturally without the aid of ART. Additionally, 44.23% (n=46) had at least one to three trials, while 32.69% (n=34) never had an IVF trial. It was found that 50.96% (n=53) performed their last IVF cycle more than a year ago. Results showed that 25% (n=26) of the enrolled women were infected with COVID-19, and 42.31% (n=44) had an infected family member.

**Table 1:** Socio-demographic characteristics of the study participants (n=104)

Variable	n (%)
Age	
18–24	7 (6.73)
25–31	36 (34.61)
32–39	49 (47.12)
40 years or more	12 (11.54)
Level of education	
Less than high school	14 (13.46)
High school	27 (25.96)
College	58 (55.77)
Postgraduate	5 (4.81)
Cause of infertility	
Female	24 (23.08)
Male	22 (21.15)
Both	13 (12.5)
Unexplained	45 (43.27)
Did you ever get pregnant before?	
Yes	57 (54.81)
No	47 (45.19)
If yes, was it with the aid of ART?	
Yes	24 (23.07)
No	33 (31.73)
Number of previous ART attempts	
1–3 trials	46 (44.23)
4–6 trials	16 (15.39)
6–10 trials	8 (7.69)
No trials	34 (32.69)
When was your last IVF cycle?	
Less than 3 months ago	18 (17.31)
3–6 months ago	16 (15.38)
6–12 months ago	17 (16.35)
More than a year ago	53 (50.96)
Did you get infected with COVID-19?	
Yes	26 (25.00)
No	78 (75.00)
Did anyone in your family get infected with COVID-19?	
Yes	44 (42.31)
No	60 (57.69)

The psychological stress scale is demonstrated in Table 2. Surprisingly, 61 women reported that the COVID-19 pandemic did not affect their mental health or affected less than expected, while 33 found it as stressful as expected. Moreover, 19.23% (n=20) were in the middle of an IVF cycle while lockdown was mandated. Stress response to IVF clinic closure was reported as follows: 31.73% (n=33) as expected, 38.46% (n=40) less than expected, and 29.81% (n=31) higher than expected. The majority of women sought the ART clinic directly after reopening, accounting for 65.38% (n=68), and reported no menstrual cycle changes during the pandemic (89.42%, n=93). The desire to conceive was affected less than expected during COVID-19 (68.62%, n=71). Stress due to delay in pregnancy plans was found to be 27.88% (n=29) as expected, 43.27% (n=45) less than expected, and 28.85% (n=30) higher than expected. One to two IVF trials had the highest number of cycles after reopening, 80.76% (n=84). Only 8.66% (n=9) achieved pregnancy after lockdown with the aid of ART. Most women found it relieving that a vaccine was developed (86.54%, n=90), yet only 50.96% (n=51) were vaccinated.

**Table 2:** Psychological stress scale responses (n=104)

Variable	n (%)
Did home quarantine affect your mental health?	
As expected	33 (31.73)
Not at all or less than expected	61 (58.65)
Slightly higher than expected or higher than usual	10 (9.62)
Were you in the middle of an IVF cycle during the clinic shutdown due to the pandemic?	
Yes	20 (19.23)
No	84 (80.77)
Did the closure of ART clinics cause you any stress?	
As expected	33 (31.73)
Not at all or less than expected	40 (38.46)
Slightly higher than expected or higher than usual	31 (29.81)
Did the pandemic affect your desire to get pregnant?	
As expected	20 (19.24)
Not at all or less than expected	71 (68.26)
Slightly higher than expected or higher than usual	13 (12.5)
Did you feel stressed regarding the delay in pregnancy plans during the pandemic?	
As expected	29 (27.88)
Not at all or less than expected	45 (43.27)
Slightly higher than expected or higher than usual	30 (28.85)
Was there any change in your sleep during the pandemic?	
Not at all	83 (79.81)
Severely disturbed	18 (17.31)
Slightly disturbed	3 (2.88)
Was there a change in your eating habits during the pandemic?	
Not at all	52 (50)
Slight effect	40 (38.46)
Major effect	12 (11.54)
Were there any changes in the regularity of the menstrual cycle during the pandemic?	
Yes	11 (10.58)
No	93 (89.42)
Did you have support from your family/close friends during the pandemic in regard to your pregnancy attempts?	
Yes	68 (65.38)
No	36 (34.62)
If yes, did it have a positive impact on your mental health?	
Yes	80 (76.92)
No	24 (23.08)
Did you think of visiting a psychiatrist/ psychologist during the pandemic?	
Yes	19 (18.27)
No	85 (81.73)
Did you visit infertility clinics seeking ART post reopening?	
Yes	68 (65.38)
No	36 (34.62)
The number of cycles that you had post re-opening?	
1–2 cycles	84 (80.76)
3–4 cycles	10 (9.62)
5–6 cycles	10 (9.62)
Did you achieve pregnancy post-COVID-19 using ART?	
Yes	9 (8.66)
No	95 (91.34)
Did you feel a sense of relief post-COVID-19 vaccine development?	
Yes	90 (86.54)
No	14 (13.46)
Did you receive COVID-19 vaccine?	
Yes	51 (49.04)
No	53 (50.96)

Table 3 depicts the levels of psychological stress. The majority of women had severe stress levels, accounting for 39.42% (n=41). The overall prevalence of psychological stress accounted for 86.5% (n=90).

**Table 3:** Levels of psychological stress (n=104)

Score	n (%)
Mild	14 (13.46)
Moderate	36 (34.62)
Severe	41 (39.42)
Extremely affected	13 (12.50)

In Table 4, an association between psychological stress levels and sample demographics. The number of previous ART trials was the only statistically significant variable (p=0.0019).

**Table 4:** Association between the sample demographics and stress level

Variables	(n)%	Score				P value
		Mild	Moderate	Severe	Extremely severe	
Age						
18-24	7 (6.73)	-	2 (5.56)	2 (4.88)	3 (23.08)	0.7292
25-31	36 (34.62)	5 (35.71)	12(33.33)	15 (36.59)	4 (30.77)	
32-39	49 (47.12)	7 (50.00)	19 (52.78)	18 (43.90)	5 (38.46)	
Above 40	12 (11.54)	2 (14.29)	3 (8.33)	6 (14.63)	1 (7.69)	
Level of education						
College	58 (55.77)	7 (50.00)	21 (58.33)	25 (60.98)	5 (38.46)	0.5556
High school	27 (25.96)	5 (35.71)	7 (19.44)	9 (21.95)	6 (46.15)	
Less than high school	14 (13.46)	2 (14.29)	7 (19.44)	4 (9.76)	1 (7.69)	
Post-graduate	5 (4.81)	-	1 (2.78)	3 (7.32)	1 (7.69)	
Cause of infertility						
Both	13 (12.50)	1 (7.14)	6 (16.67)	6 (14.63)	-	0.5609
Female	24 (23.08)	4 (28.57)	7 (19.44)	8 (19.51)	5 (38.46)	
Male	22 (21.15)	5 (35.71)	5 (13.89)	9 (21.95)	3 (23.08)	
Unexplained	45 (43.27)	4 (28.57)	18 (50.00)	18 (43.90)	5 (38.46)	
Did you ever get pregnant before?						
No	47 (45.19)	6 (42.86)	17 (47.22)	17 (41.46)	7 (53.85)	0.8734
Yes	57 (54.81)	8 (57.14)	19 (52.78)	24 (58.54)	6 (46.15)	
If yes, was it with the aid of ART?						
No	38 (57.58)	6 (75.00)	10 (43.48)	17 (62.96)	5 (62.50)	0.3773
Yes	28 (42.42)	2 (25.00)	13 (56.52)	10 (37.04)	3 (37.50)	
Number of previous ART attempts?						
1-3 trial	46 (44.66)	5 (35.71)	23 (65.71)	14 (34.15)	4 (30.77)	0.0019
4-6 trial	16 (15.53)	-	8 (22.86)	6 (14.63)	2 (15.38)	
6-10 trials	7 (6.80)	2 (14.29)	-	5 (12.20)	-	
No trials	34 (33.01)	7 (50.00)	4 (11.43)	16 (39.02)	7 (53.85)	
When was your last IVF cycle?						
6-12 months	13 (15.66)	1 (11.11)	3 (10.00)	8 (24.24)	1 (9.09)	0.6170
3-6 months	12 (14.46)	1 (11.11)	7 (23.33)	2 (6.06)	2 (18.18)	
Less than 3 months	14 (16.87)	1 (11.11)	5 (16.67)	5 (15.15)	3 (27.27)	
More than a year	44 (53.01)	6 (66.67)	15 (50.00)	18 (54.55)	5 (45.45)	
Did you get infected with COVID-19?						
No	78 (75.00)	12 (85.71)	27 (75.00)	31 (75.61)	8 (61.54)	0.5645
Yes	26 (25.00)	2 (14.29)	9 (25.00)	10 (24.39)	5 (38.46)	
Did anyone in your family get infected with COVID-19?						
No	60 (57.69)	8 (57.14)	23 (63.89)	23 (56.10)	6 (46.15)	0.7277
Yes	44 (42.31)	6 (42.86)	13 (36.11)	18 (43.90)	7 (53.85)	

## Discussion

A key question within the field of mental health sciences is how we are going to cope with and adapt to the likely mental health impact of COVID-19 among the population. The mental health community has mobilized with incredible speed and efficiency to ensure that the support that is required currently is available. Pregnancy-seeking women were among the populations that received the attention of researchers in terms of the effect of COVID-19 on their mental health. However, women who attend ART clinics have received little attention compared to other vulnerable groups that are significantly affected by the COVID-19 pandemic [13]. The present study sought to explore the prevalence of psychological stress and its levels among Saudi women attending the ART clinics at the Reproductive Medicine Department at King Abdulaziz Medical City in Riyadh City (KAMC-RD).

Our findings revealed that moderate (34.62%) and severe (39.42%) psychological stress were the highest prevalent among the enrolled women, which is an alarming situation that could be referred to the precautionary measures taken due to the COVID-19 pandemic and limit the daily life activities of the individuals, especially women whose their daily activities included seeking pregnancy through the aid of ART. Different studies, such as Rooney et al. [14], reported that there is a significant association between infertility and stress due to overthinking and the concern of the treatment outcome reaching up to 40% [10-12]. Nevertheless, this stress might be exacerbated due to the pandemic, especially since women are unable to seek help through any means. These elevated stress levels might be attributed to that a number of the enrolled women were in the middle of the IVF cycle (19.23%), and there was a lack of support from either relatives or family members (34.62%) [2-3,15].

Furthermore, the increased prevalence of psychological stress among women attending IVF clinics might be attributed to the high number of women who did not achieve pregnancy post-COVID-19 using ART (91.34%), which imposed higher pressure and stress on the women, especially since this study is performed within a middle-eastern community that strongly tied to the beliefs, indicating the necessity of the woman to get pregnant as soon and as many as possible [7-11].

Our findings revealed that there is a significant statistical interaction between the number of previous ART attempts and the levels of psychological stress among the enrolled women. This finding might be referred to the woman's fear of failure to have children, especially since her chances of having children decrease with age, which will negatively affect her relationship with her husband and change the view of those around her, especially in a society that considers the necessity of childbearing early in marriage to avoid negative criticism of the wife from those around her.

Despite the significant findings reported in the present study, still there are a number of limitations that might limit the generalization of the study findings. These limitations include the low sample size, which could affect the validity and reliability of the study findings. Another limitation is being a single-center study, as this study was performed in a single setting. Therefore, the findings of the study might not be applicable to other settings. The study findings are limited due to the psychometric properties of the data collection tool.

## Conclusions

All in all, there is increasing psychological stress among women attending IVF clinics in our center during the COVID-19 pandemic. We found a significant interaction between the number of previous ART attempts and the level of psychological stress among the study sample. Based on the aforementioned findings, we urge multi-centered studies with higher levels of evidence to investigate the relationship between stress and success rates.
